# Comparison of GLP-1 Receptor Agonists, SGLT-2 Inhibitors, and DPP-4 Inhibitors as an Add-On Drug to Insulin Combined With Oral Hypoglycemic Drugs: Umbrella Review

**DOI:** 10.1155/2024/8145388

**Published:** 2024-07-20

**Authors:** Sanbao Chai, Yapin Niu, Fengqi Liu, Shanshan Wu, Zhirong Yang, Feng Sun

**Affiliations:** ^1^ Department of Endocrinology and Metabolism Peking University International Hospital, Beijing 102206, China; ^2^ Department of Epidemiology and Biostatistics Peking University School of Public Health, Beijing 100083, China; ^3^ National Clinical Research Center of Digestive Diseases Capital Medical University Affiliated Beijing Friendship Hospital, Beijing 100050, China; ^4^ Shenzhen Institute of Advanced Technology Chinese Academy of Sciences, Shenzhen 518055, China; ^5^ Key Laboratory of Epidemiology of Major Disease (Peking University) Ministry of Education, Beijing 100191, China

**Keywords:** basic insulin, DPP-4, GLP-1, SGLT-2, Type 2 diabetes, umbrella review

## Abstract

**Background:** The objective was to evaluate the efficacy of the combination of Glucagon-like peptide-1 receptor agonists (GLP-1 RAs), dipeptidyl peptidase-4 inhibitors (DPP-4i), and sodium-glucose cotransporter 2 inhibitor (SGLT-2i) in the treatment of Type 2 diabetes with poor efficacy of basic insulin and metformin/sulfonylurea by umbrella review.

**Materials and Methods:** Forming the data of publication of each database through 13 September 2022, PubMed, EMBASE, and Cochrane Library were surveyed.

**Results:** A total of seven meta-analyses were included in the umbrella review. The combination of GLP-1 RA (WMD −3.41 [−5.61, −1.21], *p* = 0.002), SGLT-2i (WMD −5.34 [−9.56, −1.13], *p* = 0.013), and DPP-4i (WMD −5.56 [−7.39, −3.73], *p* ≤ 0.001) can significantly reduce HbA1c levels, respectively. The combination of GLP-1 RA (WMD −1.55 [−2.92, −0.18], *p* = 0.027), SGLT-2i (WMD −2.96 [−6.68, 0.77], *p* = 0.12), and DPP-4i (WMD −2.05 [−2.82, −1.28], *p* ≤ 0.001) can significantly reduce fasting plasma glucose (FPG) levels, respectively. The combination of GLP-1 RA (WMD −3.24 [−5.14, −1.34], *p* < 0.001) can significantly reduce body weight of Type 2 diabetes mellitus (T2DM). The dose of basic insulin in diabetes patients after combined use of GLP-1 RA (WMD −2.74 [−4.26, −1.22], *p* ≤ 0.001) was significantly reduced. The combination use of GLP-1 RAs (OR 1.28 [1.05, 1.56], *p* = 0.017) increases the risk of hypoglycemia.

**Conclusions:** The combination of GLP-1 RAs, DPP-4i, and SGLT-2i can effectively lower HbA1c and FPG in T2DM patients who have poor therapeutic effects on basic insulin combined with metformin/sulfonylureas, respectively. Compared to placebo, GLP-1 RAs can significantly reduce body weight and basic insulin dosage, while DPP-4i and SGLT-2i have a lower risk of hypoglycemia.

**Trial Registration:** CRD42023410345.

## 1. Introduction

Type 2 diabetes mellitus (T2DM) is a chronic metabolic disorder characterized by a relentless deterioration in insulin sensitivity and a subsequent, progressive decline in pancreatic beta-cell function. Impaired beta-cell function is a recognized cornerstone of diabetes pathophysiology. This ultimately leads to a sustained elevation of blood sugar levels (hyperglycemia). Long-term hyperglycemia and its associated complications are metabolic diseases with high morbidity that result in poor quality of health and life. Therefore, most T2DM patients ultimately need to initiate insulin treatment to control blood sugar, and the principle of insulin treatment is to simulate physiological insulin secretion and action as much as possible. Among numerous insulin preparations, basic insulin has become the first recommended initial insulin treatment plan due to its low daily injection frequency, coverage of 24 h, convenient use, and low risk of hypoglycemia [1–3]. However, as the disease progresses, basic insulin combined with metformin or sulfonylurea drugs cannot maintain glycated hemoglobin at 7%. The combination therapy of multiple hypoglycemic drugs is imperative. Glucagon-like peptide-1 receptor agonists (GLP-1 RAs) have been shown to improve glycemic control and aid weight loss, with a lower risk of hypoglycemia compared with other hypoglycemic drugs. With the results from cardiovascular outcomes trials, they are one of the preferred drugs for patients with T2DM and established atherosclerotic cardiovascular disease (ASCVD) [4]. Sodium-glucose cotransporter 2 inhibitors (SGLT-2i) can enhance weight loss, concomitantly lowering HbA1c, reducing the combined risk of cardiovascular death or the risk of renal events and hospitalization for heart failure [5, 6].

Dipeptidyl peptidase-4 inhibitors (DPP-4i) have been available for treating T2DM owing to their good safety profile and tolerability, as well as their efficacy in improving glycemic control [7]. How to combine other types of hypoglycemic drugs in the future? And how to use these hypoglycemic drugs in different diabetes patients is a problem that clinicians often need to face. Only by objectively evaluating the combined hypoglycemic drugs can reasonable recommendations be made on the benefits and risks of the drugs.

Umbrella review, also known as umbrella evaluation, is an internationally emerging review and evaluation method in recent years. It can re-evaluate the previously published systematic review and meta-analysis (SR/MA), summarize the evidence from various studies, and obtain reliable conclusions [8].

The purpose of this study was to compare the efficacy and safety of GLP-1 RAs, DPP-4i, and SGLT-2i in combination with basic insulin and oral hypoglycemic agents in poorly treated T2DM through umbrella review, respectively.

## 2. Materials and Methods

### 2.1. Search Strategy and Literature Search

We conducted a review of literatures from online databases of literatures including PubMed, Cochrane Library, and EMBASE. The date ranges from publication of each database through 13 September 2022. We used “type 2 Diabetes Mellitus,”“Basic Insulin,” “Metformin,” “Sulfonylureas,” “Systematic Review,” and “Meta-analysis” as keywords or MeSH/EMTREE terms, accompanied with other relevant free words to search these databases. Details of the search strategy are provided in supplementary materials (Table S1).

### 2.2. Literature Screening and Selection

The subjects were T2DM who were poorly treated with basic insulin combined with metformin/sulfonylureas and were subsequently combined with GLP-1 RAs, DPP-4i, or SGLT-2i, respectively. All included studies are MA based on clinical randomized controlled trial. The eligibility of studies was assessed independently by three reviewers (YPN, SBC, and FS), with any disagreement was resolved by consensus.

### 2.3. Data Extraction

Use Excel for data extraction and management, and use preset data extraction tables to extract data from included studies. The data extraction mainly includes two parts: the included MA and the randomized control trial (RCT) information related to the purpose of this study in MA.

### 2.4. Quality Assessment

Perform AMSTAR 2 [9] quality evaluation, PRISMA 2020 [10], and Grading of Recommendations Assessment, Development and Evaluation (GRADE) evidence grading [11] report quality evaluation for each included MA. Using the Cochrane literature bias risk assessment tool (ROB 1.0) [11], bias risk assessment and GRADE evidence [12] grading were performed on the included RCTs.

### 2.5. Umbrella Review

The classic SR/MA is comprehensively analyzed based on a single original study, while the umbrella review is comprehensively evaluated based on SR/MA. Therefore, the umbrella review is at the top of the evidence-based medicine pyramid, representing one of the current high-level methods of evidence synthesis [8, 13]. The evaluation system includes two effect size measurement methods, in which equivalent Hedges'g (“eG”) for continuous variables, and equivalent odds ratio (“eOR”) for two categorical variables. In accordance with the requirements of the package, RCT data contained in SR/MA is extracted and deduplicated, and umbrella review is performed in a fixed format in strict accordance with the requirements. *I*^2^ is used to measure heterogeneity. If there is no heterogeneity or the heterogeneity is relatively small (*I*^2^ ≤ 50%), a fixed effects model is used to calculate the combined effects. On the contrary, if the heterogeneity is relatively large (*I*^2^ > 50%), a random effects model is used to merge the effect quantities. Conduct a descriptive analysis of the included SRs.

## 3. Results

### 3.1. Literature Search

**Figure 1:**
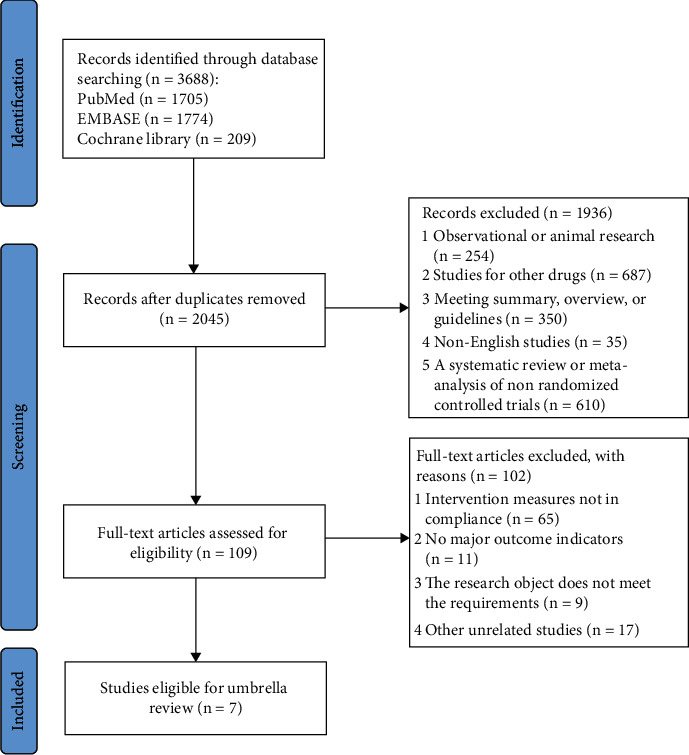
Flow chart of studies considered for inclusion.

This study was registered on the International Prospective Register of Systematic Reviews (PROSPERO), and the registration number is CRD42023410345. Our search strategy resulted in the identification of 3688 articles (Figure 1). After the extensive review of the titles and abstracts of these articles, 109 articles were identified for full-text review. Of these, seven studies were finally included in our umbrella review.

### 3.2. Study Characteristics

A total of seven meta-analyses [14–20] were included in the umbrella review, including 58 RCTs with a total of 18,786 patients. Detailed information of seven studies is shown in Table 1.

### 3.3. Methodological Quality Evaluation

#### 3.3.1. AMSTAR 2 Scale Evaluation

The AMSTAR 2 scale was used to evaluate the methodological quality of seven articles. Five articles are of high quality, while the other two articles are of medium and low quality, respectively (Figure S1).

#### 3.3.2. PRISMA 2020 Evaluation

The PRISMA scores included in the study range from 21.5 to 26, and the information reported in the literature is relatively complete (Figure S2).

### 3.4. Umbrella Review Results

#### 3.4.1. Changes of HbA1c

There were 17,957 T2DM patients which had HbA1c level as the outcome indicator in 56 RCTs.

**Figure 2:**
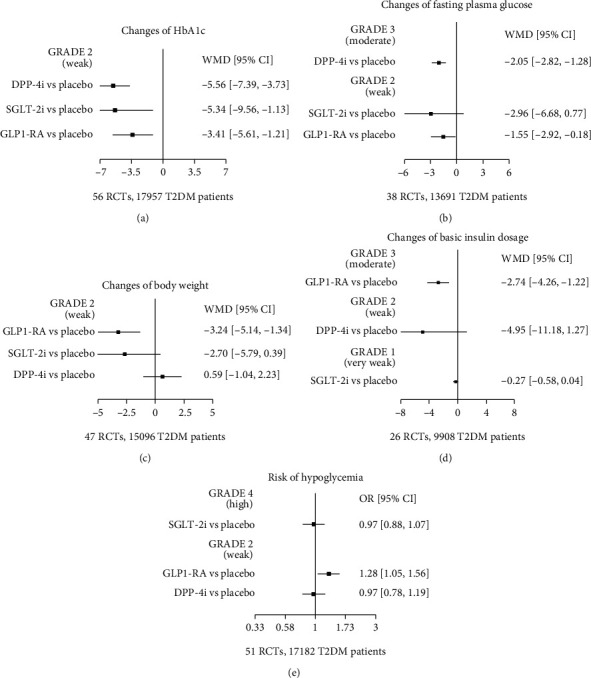
Umbrella review results: (a) changes of HbA1c; (b) changes of fasting plasma glucose; (c) changes of body weight; (d) changes of basic insulin dosage; (e) risk of hypoglycemia. Placebo: basic insulin combined with metformin/sulfonylureas.

Compared to the original treatment, the combination of GLP-1 RA (WMD −3.41 [−5.61, −1.21], *p* = 0.002), SGLT-2i (WMD −5.34 [−9.56, −1.13], *p* = 0.013), and DPP-4i (WMD −5.56 [−7.39, −3.73], *p* ≤ 0.001) can significantly reduce HbA1c levels, respectively (Figure 2(a)).

#### 3.4.2. Changes of Fasting Plasma Glucose (FPG)

There were 13,691 T2DM patients which had FPG level as the outcome indicator in 38 RCTs. Compared to the original treatment, the combination of GLP-1 RA (WMD −1.55 [−2.92, −0.18], *p* = 0.027), SGLT-2i (WMD −2.96 [−6.68, 0.77], *p* = 0.12), and DPP-4i (WMD −2.05 [−2.82, −1.28], *p* ≤ 0.001) can significantly reduce FBG levels, respectively (Figure 2(b)).

#### 3.4.3. Changes of Body Weight

There were 15,096 T2DM patients which had body weight as the outcome indicator in 47 RCTs. Compared to the original treatment, the combination of GLP-1 RA (WMD −3.24 [−5.14, −1.34], *p* < 0.001) can significantly reduce body weight of T2DM. Compared to the original treatment, the combination of SGLT-2i (WMD −2.70 [−5.79, 0.39], *p* = 0.087) and DPP-4i (WMD 0.59 [−1.04, 2.23], *p* = 0.476) did not affect body weight of T2DM, respectively (Figure 2(c)).

#### 3.4.4. Changes of Basic Insulin Dosage

There were 9908 T2DM patients which had basic insulin dosage as the outcome indicator in 26 RCTs. Compared to the original treatment, the dose of basic insulin in diabetes patients after combined use of GLP-1 RA (WMD −2.74 [−4.26, −1.22], *p* ≤ 0.001) was significantly reduced. Compared to the original treatment, the dose of basic insulin in diabetes patients did not change treated with SGLT-2i (WMD −0.27 [−0.58, 0.04], *p* = 0.086) and DPP-4i (WMD −4.95 [−11.18, 1.27], *p* = 0.119), respectively (Figure 2(d)).

#### 3.4.5. Risk of Hypoglycemia

There were 17,182 T2DM patients which had hypoglycemia as the outcome indicator in 51 RCTs.

Compared to the original treatment, the combination use of GLP-1 RAs (OR 1.28 [1.05, 1.56], *p* = 0.017) increases the risk of hypoglycemia. Compared to the original treatment, the combination of SGLT-2i (OR 0.97 [0.88, 1.07], *p* = 0.548) and DPP-4i (OR 0.97 [0.78, 1.19], *p* = 0.744) did not increase the risk of hypoglycemia, respectively (Figure 2(e)).

## 4. Discussion

With the progression of Type 2 diabetes, the function of pancreatic islets gradually decreases, and the blood sugar of patients gradually increases. The strategy of hypoglycemic treatment needs to be constantly adjusted. The American Diabetes Association (ADA) issued Pharmacologic Approaches to Glycemic Treatment: Standards of Care in Diabetes-2024 [21], which pointed out that initiation of insulin should be considered regardless of background glucose lowering therapy or disease stage if symptoms of hyperglycemia are present, or when A1C is very high (> 10%). If the combined treatment with basic insulin still fails, another drug such as SGLT-2i and GLP-1RA can be added.

However, the complications of diabetes patients, the risk of hypoglycemia, and the impact on weight should be considered when selecting the above drugs. Therefore, it is necessary to evaluate the hypoglycemic efficacy and safety of the aforementioned drugs.

In recent years, umbrella review has been used to evaluate the effect of diet and information management on blood glucose in patients with T2DM [22, 23]. The results of the meta-analysis of RCT and the umbrella review containing 26 meta-analyses support the theory that increasing microbiota-accessible carbohydrate intake can improve the cardiac metabolic risk factors of T2DM and has advantages in the diet management of T2DM [18]. A recently published article included 95 meta-analyses to analyze the risk of patients with prediabetes by umbrella review [24]. The results suggest that prediabetes was positively associated with risk of all-cause mortality and the incidence of cardiovascular outcomes, CHD, stroke, chronic kidney disease, cancer, and dementia [24]. In addition, the published umbrella review provides evidence on how to reduce the risk of type 2 diabetes through diet and how diabetes patients manage their weight through reasonable diet [25, 26], thus providing a basis for clinicians and nutritionists to formulate strategies.

The goals of treatment for T2DM are to prevent or delay complications and optimize quality of life. So Type 2 diabetes patients with established high risk or ASCVD, heart failure, and/or chronic kidney disease, the hypoglycemic regimen should choose GLP-1 RAs or SGLT-2i. Patients with T2DM often have osteoporosis, which increases the risk of fracture. Clinical trials and postmarketing data both indicate that DPP-4i and GLP-1 RAs have a neutral impact on bone health [27, 28]. For Type 2 diabetes patients with osteoporosis, the use of DPP-4i and GLP-1 RAs for hypoglycemic treatment will not affect bone metabolism. The results of this study suggest that GLP-1 RA not only has a significant hypoglycemic effect but also can reduce insulin dosage. In patients with poor glycemic control of basic insulin and metformin/sulfonylurea, the subsequent combined with SGLT-2i or DPP-4i has the same significant hypoglycemic effect. Meanwhile, there is no risk of hypoglycemia. Therefore, evaluating hypoglycemic drugs can provide individualized treatment plans for doctors, maximizing the clinical benefits of patients with T2DM.

The advantage of this study is that the umbrella review can automatically fit multiple meta-analyses, re-evaluate existing SR/MA, automatically extract necessary information to score the evidence, and automatically evaluate publication bias. Therefore, it effectively obtains relatively objective results and has certain guiding significance for doctors to choose hypoglycemic drugs based on individual patient conditions.

The management of diabetes should be guided by the joint decision of evaluating the patients' overall health status, diabetes complications, cardiovascular risks, hypoglycemia risks, and treatment goals. Therefore, GLP-1 RAs and SGLT-2i are the first choice for combined treatment of Type 2 diabetes patients with cardiovascular disease or with cardiovascular risk factors, chronic kidney disease, and heart failure. For elderly patients with diabetes who are at high risk of fracture, GLP-1 RAs and DPP-4i can be selected for combined treatment. In conclusion, type 2 diabetes is a progressive disease, and it usually requires combined treatment to maintain the blood glucose level. The purpose of combination therapy should be patient centered, while also bringing multiple benefits.

There are also some shortcomings in this study. Firstly, this study only considers the inclusion of English literature, which may lead to some publication bias in the results of this study. Secondly, the evaluation of subsequent treatment plans did not consider the impact of drug dosage and course of treatment, mainly due to the limited number of dose combinations reported in the original literature, which poses significant difficulties in constructing dose-response relationships. Thirdly, the impact on cardiovascular outcomes was not considered in the evaluation of subsequent treatment, mainly due to incomplete reporting on cardiovascular outcomes in the included literature and limited data on indicators for analyzing cardiovascular events.

## 5. Conclusions

The combination of GLP-1 RAs, DPP-4i, and SGLT-2i can effectively lower HbA1c and FPG in T2DM patients who have poor therapeutic effects on basic insulin combined with metformin/sulfonylureas, respectively. Compared with placebo, GLP-1 RAs can significantly reduce body weight and basic insulin dosage, while DPP-4i and SGLT-2i have a lower risk of hypoglycemia.

## Figures and Tables

**Table 1:** Study characteristics of included studies.

**Study ID**	**SR/MA**	**Number of RCT**	**Number of patients (treatment/control)**	**Background medicine**	**Intervention measures**	**Control**	**Outcome indicators**	**Bias of estimation**
Kim et al., 2016 [14]	MA	9	2306/2158	Basic insulin+metformin	DPP-4i^b^	Basic insulin+metformin	①②③④⑤	Cochrane
Maiorino et al., 2017 [15]	MA	26	5689/5736	Basic insulin+metformin/sulfonylureas	GLP-1 RA^c^	Basic insulin+metformin	①③⑤	Cochrane
Min et al., 2017 [16]	MA	14	3561/3419	Basic insulin+metformin/sulfonylureas	DPP-4i^d^	Basic insulin+metformin		Cochrane
					SGLT-2i^e^	Basic insulin+metformin	①②③④⑤	
Cho et al., 2018 [17]	MA	14	2938/4288	Basic insulin+pioglitazone	SGLT-2i^f^	Basic insulin+metformin	①②③④⑤	Cochrane
Yang et al., 2018 [18]	MA	36	3241/2915	Basic insulin+metformin/OADs^a^	DPP-4i^g^	Basic insulin+metformin	①②③④⑤	Cochrane
					GLP-1 RA^h^	Basic insulin+metformin		
Yoon et al., 2018 [19]	MA	50	8753/6741	Basic insulin±metformin	DPP-4i^i^	Basic insulin+metformin	①②③④⑤	Cochrane
					GLP-1 RA^j^	Basic insulin+metformin		
					SGLT-2i^k^	Basic insulin+metformin		
Maiorino et al., 2019 [20]	MA	36	7041/7595	Basic insulin+metformin/sulfonylureas	GLP-1 RA^l^	Basic insulin+metformin	①③⑤	Cochrane

*Note:* ① Changes in HbA1c. ② Change of fasting plasma glucose. ③ Weight change. ④ Changes in insulin dosage. ⑤ Incidence of hypoglycemic events.

Abbreviations: DPP-4i: dipeptidyl peptidase-4 inhibitors; GLP-1 RA: glucagon-like peptide-1 receptor agonist; MA: meta-analysis; SGLT-2i: sodium glucose cotransporter-2 inhibitors; SR: systematic review.

^a^Oral hypoglycemic drugs (placebo; alpha-glucosidase inhibitors; thiazolidinedione).

^b^Saxagliptin 5 mg QD, vildagliptin 50 mg BID, sitagliptin 50 mg QD, alogliptin 25 mg QD, vildagliptin 50 mg BID, sitagliptin 100 mg QD, alogliptin 25 mg QD, sitagliptin 100 mg QD, and linagliptin 5 mg QD.

^c^Exenatide 10 *μ*g BID, lixisenatide 20 *μ*g QD, liraglutide 1.8 mg QD, liraglutide 0.9 mg QD, liraglutide 1.2 mg QD, albiglutide 30 mg QW, dulaglutide 1.5 mg QW, and exenatide 5–10 *μ*g BID.

^d^Saxagliptin 5 mg QD, vildagliptin 50 mg BID, sitagliptin 50 mg QD, alogliptin 25 mg QD, vildagliptin 50 mg BID, sitagliptin 100 mg QD, and linagliptin 5 mg QD.

^e^Canagliflozin 300 mg QD, empagliflozin 25 mg QD, and dapagliflozin 10 mg QD.

^f^Dapagliflozin 10 mg QD, dapagliflozin 5 mg QD, empagliflozin 10 mg QD, empagliflozin 25 mg QD, canagliflozin 100 mg QD, canagliflozin 300 mg QD, and ipragliflozin 50 mg QD.

^g^Unspecified.

^h^Unspecified.

^i^Saxagliptin 5 mg QD, vildagliptin 50 mg BID, sitagliptin 50 mg QD, alogliptin 25 mg QD, sitagliptin 100 mg QD, sitagliptin 50 or 100 mg QD, and linagliptin 5 mg QD.

^j^Liraglutide 0.6–1.8 mg QD, exenatide 10 *μ*g BID, lixisenatide 10–20 *μ*g QD, lixisenatide 20 *μ*g QD, liraglutide 0.9 mg QD, and dulaglutide 1.5 mg QW.

^k^Dapagliflozin 5 mg QD, dapagliflozin 10 mg QD, canagliflozin 100 mg QD, canagliflozin 300 mg QD, and empagliflozin 25 mg QD.

^l^Exenatide 10 *μ*g BID, liraglutide 1.8 mg QD, liraglutide 1.2 mg QD, lixisenatide 20 *μ*g QD, liraglutide 0.9 mg, dulaglutide 1.5 mg QW, semaglutide 0.5 mg QW, semaglutide 1 mg QW, lixisenatide 20 *μ*g QD, albiglutide 30 mg QW, and dulaglutide 0.75 mg QW.

## Data Availability

Data can be available on reasonable request.
