# Supplementary material for: Comparison of GLP-1 Receptor Agonists, SGLT-2 Inhibitors, and DPP-4 Inhibitors as an Add-On Drug to Insulin Combined With Oral Hypoglycemic Drugs: Umbrella Review
Source: J Diabetes Res. 2024 Jul 20;2024:8145388. doi: 10.1155/2024/8145388 (PMC11283333; doi:10.1155/2024/8145388)
Supplement: Supporting Information — Additional supporting information can be found online in the Supporting Search strategy, AMSTAR 2 quality evaluation, and PRISMA 2020 evaluation. [file 8145388.f1.zip › Supplementary Table 1.docx]

Supplementary Table 1 Search strategy

PubMed

| **Order** | **Search terms** |
| --- | --- |
| #1 | "diabetes mellitus, type 2"[MeSH Terms] |
| #2 | "type 2 diabetes mellitus"[Title/Abstract] OR "type ii diabetes mellitus"[Title/Abstract] OR "NIDDM"[Title/Abstract] OR "maturity onset diabetes"[Title/Abstract] OR "adult onset diabetes mellitus"[Title/Abstract] OR "ketosis resistant diabetes mellitus"[Title/Abstract] OR "non insulin dependent diabetes mellitus"[Title/Abstract] OR (("stable"[All Fields] OR "stabled"[All Fields] OR "stables"[All Fields] OR "stabling"[All Fields]) OR "diabetes mellitu"[Title/Abstract]) OR "maturity onset diabetes mellitus"[Title/Abstract] OR "MODY"[Title/Abstract] |
| #3 | "Metformin"[Text Word] OR "sitagliptin phosphate"[Text Word] OR "Glucophage"[Text Word] OR "Sulfonylureas"[Text Word] OR "Glibenclamide"[Text Word] OR (("Glyburide"[MeSH Terms] OR "Glyburide"[All Fields] OR "hb 419"[All Fields]) OR "Maninil"[Text Word]) OR "Adiab"[Text Word] OR "Glyburide"[Text Word] OR "glybenzcyclamide"[Text Word] OR "glybenzcyclamide"[Text Word] OR "euglucan"[Text Word] OR "Micronase"[Text Word] OR "USAN"[Text Word] OR "INN"[Text Word] OR "Glucovance"[Text Word] OR (("glibenclamid"[All Fields] OR "Glyburide"[MeSH Terms] OR "Glyburide"[All Fields] OR "Glibenclamide"[All Fields] OR "glyburide s"[All Fields]) OR "usp"[Text Word]) OR "Gliclazide"[Text Word] OR "Diamicron"[Text Word] OR "Glipizide"[Text Word] OR "Glibenese"[Text Word] OR "Gliquidone"[Text Word] OR "Gliquidon"[Text Word] OR "glimepiride"[Text Word] OR "Amaryl"[Text Word] OR "Hoe-490"[Text Word] OR ("Insulin"[Text Word]) OR "NPH"[Text Word] OR "neutral protamine hagedorn"[Text Word] OR "Ultralente"[Text Word] OR "protamine zinc insulin"[Text Word] OR "PZI"[Text Word] OR "lente"[Text Word] OR "Glargine"[Text Word] OR "Lantus"[Text Word] OR "Detemir"[Text Word] OR "Levemir"[Text Word] OR "Degludec"[Text Word] OR "Tresiba"[Text Word] |
| #4 | "meta-analysis"[Publication Type] OR "systematic review"[Publication Type] |
| #5 | "diabetes mellitus, type 2"[MeSH Terms] OR ("type 2 diabetes mellitus"[Title/Abstract] OR "type ii diabetes mellitus"[Title/Abstract] OR "NIDDM"[Title/Abstract] OR "maturity onset diabetes"[Title/Abstract] OR "adult onset diabetes mellitus"[Title/Abstract] OR "ketosis resistant diabetes mellitus"[Title/Abstract] OR "non insulin dependent diabetes mellitus"[Title/Abstract] OR (("stable"[All Fields] OR "stabled"[All Fields] OR "stables"[All Fields] OR "stabling"[All Fields]) OR "diabetes mellitu"[Title/Abstract]) OR "maturity onset diabetes mellitus"[Title/Abstract] OR "MODY"[Title/Abstract]) |
| #6 | ("Metformin"[Text Word] OR "sitagliptin phosphate"[Text Word] OR "Glucophage"[Text Word] OR "Sulfonylureas"[Text Word] OR "Glibenclamide"[Text Word] OR (("Glyburide"[MeSH Terms] OR "Glyburide"[All Fields] OR "hb 419"[All Fields]) OR "Maninil"[Text Word]) OR "Adiab"[Text Word] OR "Glyburide"[Text Word] OR "glybenzcyclamide"[Text Word] OR "glybenzcyclamide"[Text Word] OR "euglucan"[Text Word] OR "Micronase"[Text Word] OR "USAN"[Text Word] OR "INN"[Text Word] OR "Glucovance"[Text Word] OR (("glibenclamid"[All Fields] OR "Glyburide"[MeSH Terms] OR "Glyburide"[All Fields] OR "Glibenclamide"[All Fields] OR "glyburide s"[All Fields]) OR "usp"[Text Word]) OR "Gliclazide"[Text Word] OR "Diamicron"[Text Word] OR "Glipizide"[Text Word] OR "Glibenese"[Text Word] OR "Gliquidone"[Text Word] OR "Gliquidon"[Text Word] OR "glimepiride"[Text Word] OR "Amaryl"[Text Word] OR "Hoe-490"[Text Word] OR ("Insulin"[Text Word]) OR "NPH"[Text Word] OR "neutral protamine hagedorn"[Text Word] OR "Ultralente"[Text Word] OR "protamine zinc insulin"[Text Word] OR "PZI"[Text Word] OR "lente"[Text Word] OR "Glargine"[Text Word] OR "Lantus"[Text Word] OR "Detemir"[Text Word] OR "Levemir"[Text Word] OR "Degludec"[Text Word] OR "Tresiba"[Text Word]) AND ("meta-analysis"[Publication Type] OR "systematic review"[Publication Type]) AND ("diabetes mellitus, type 2"[MeSH Terms] OR ("type 2 diabetes mellitus"[Title/Abstract] OR "type ii diabetes mellitus"[Title/Abstract] OR "NIDDM"[Title/Abstract] OR "maturity onset diabetes"[Title/Abstract] OR "adult onset diabetes mellitus"[Title/Abstract] OR "ketosis resistant diabetes mellitus"[Title/Abstract] OR "non insulin dependent diabetes mellitus"[Title/Abstract] OR (("stable"[All Fields] OR "stabled"[All Fields] OR "stables"[All Fields] OR "stabling"[All Fields]) OR "diabetes mellitu"[Title/Abstract]) OR "maturity onset diabetes mellitus"[Title/Abstract] OR "MODY"[Title/Abstract])) |

Cochrane Library

| **Order** | **Search terms** |
| --- | --- |
| #1 | diabetes mellitus, type 2 |
| #2 | MeSH descriptor: [Diabetes Mellitus, Type 2] explode all trees |
| #3 | (type 2 diabetes mellitus):ti,ab,kw OR (type ii diabetes mellitus):ti,ab,kw OR (NIDDM):ti,ab,kw OR (maturity onset diabetes):ti,ab,kw OR (adult onset diabetes mellitus):ti,ab,kw |
| #4 | (ketosis resistant diabetes mellitus):ti,ab,kw OR (non insulin dependent diabetes mellitus):ti,ab,kw OR (stable diabetes mellitu):ti,ab,kw OR (maturity onset diabetes mellitus):ti,ab,kw OR (maturity onset diabetes mellitus):ti,ab,kw |
| #5 | #1 or #2 or #3 or #4 |
| #6 | ("metformin"):ti,ab,kw OR (Sitagliptin Phosphate):ti,ab,kw OR (Glucophage):ti,ab,kw OR (Sulfonylureas):ti,ab,kw OR (Glibenclamide):ti,ab,kw |
| #7 | (Adiab):ti,ab,kw OR (Glyburide):ti,ab,kw OR (glybenzcyclamide):ti,ab,kw OR (euglucan):ti,ab,kw OR (Glucovance):ti,ab,kw |
| #8 | (Gliclazide):ti,ab,kw OR (Glipizide):ti,ab,kw OR (Gliquidone):ti,ab,kw OR (glimepiride):ti,ab,kw OR (Gliquidon):ti,ab,kw |
| #9 | (isophand Insulin):ti,ab,kw OR (NPH):ti,ab,kw OR (neutral protamine hagedorn):ti,ab,kw OR (Ultralente):ti,ab,kw OR (protamine zinc insulin):ti,ab,kw |
| #10 | (PZI):ti,ab,kw OR (lente):ti,ab,kw OR (Glargine):ti,ab,kw OR (Lantus):ti,ab,kw OR (Detemir):ti,ab,kw |
| #11 | (Levemir):ti,ab,kw OR (Degludec):ti,ab,kw OR (Tresiba):ti,ab,kw |
| #12 | #6 or #7 or #8 or #9 or #10 or #11 |
| #13 | #5 and #12 |
| #14 | (meta-analysis):ti,ab,kw OR (systematic review):ti,ab,kw (Word variations have been searched) |
| #15 | #13 and #14 |

Embase

| **Order** | **Search terms** |
| --- | --- |
| #1 | non insulin dependent diabetes mellitus'/exp |
| #2 | Metformin:ti,ab,kw OR sitagliptin phosphate:ti,ab,kw OR Glucophage:ti,ab,kw OR Sulfonylureas:ti,ab,kw OR Glibenclamide:ti,ab,kw OR Glyburide:ti,ab,kw OR Glyburide:ti,ab,kw OR Maninil:ti,ab,kw OR Adiab:ti,ab,kw OR Glyburide:ti,ab,kw OR glybenzcyclamide:ti,ab,kw OR glybenzcyclamide:ti,ab,kw OR euglucan:ti,ab,kw OR Micronase:ti,ab,kw OR USAN:ti,ab,kw OR INN:ti,ab,kw OR Glucovance:ti,ab,kw OR glibenclamid:ti,ab,kw OR Glyburide:ti,ab,kw OR Glyburide:ti,ab,kw OR Glibenclamide:ti,ab,kw OR glyburides:ti,ab,kw OR usp:ti,ab,kw OR Gliclazide:ti,ab,kw OR Diamicron:ti,ab,kw OR Glipizide:ti,ab,kw OR Glibenese:ti,ab,kw OR Gliquidone:ti,ab,kw OR Gliquidon:ti,ab,kw OR glimepiride:ti,ab,kw OR Amaryl:ti,ab,kw OR Hoe-490:ti,ab,kw OR Insulin:ti,ab,kw OR NPH:ti,ab,kw OR neutral protamine hagedorn:ti,ab,kw OR Ultralente:ti,ab,kw OR protamine zinc insulin:ti,ab,kw OR PZI:ti,ab,kw OR lente:ti,ab,kw OR Glargine:ti,ab,kw OR Lantus:ti,ab,kw OR Detemir:ti,ab,kw OR Levemir:ti,ab,kw OR Degludec:ti,ab,kw OR Tresiba |
| #3 | #1 AND #2 |
| #4 | #1 AND #2 AND ([systematic review]/lim OR [meta analysis]/lim) |
