# Supplementary figures and images for: Comparison of GLP-1 Receptor Agonists, SGLT-2 Inhibitors, and DPP-4 Inhibitors as an Add-On Drug to Insulin Combined With Oral Hypoglycemic Drugs: Umbrella Review
Source: J Diabetes Res. 2024 Jul 20;2024:8145388. doi: 10.1155/2024/8145388 (PMC11283333; doi:10.1155/2024/8145388)

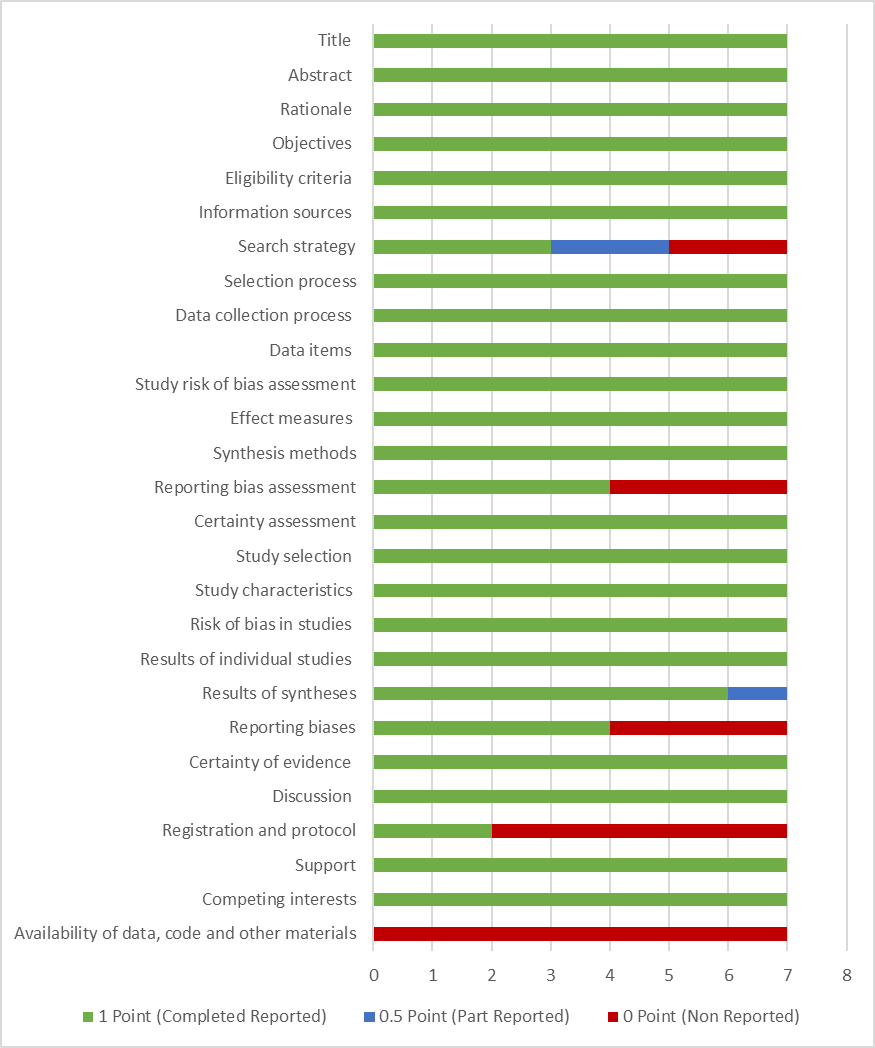


Supplementary Figure 2 PRISMA 2020 evaluation

Supplement: Supporting Information — Additional supporting information can be found online in the Supporting Search strategy, AMSTAR 2 quality evaluation, and PRISMA 2020 evaluation. [file 8145388.f1.zip › Supplementary Figure 2.docx]
